# A Dedicated Binding Mechanism for the Visual Control of Movement

**DOI:** 10.1016/j.cub.2014.02.030

**Published:** 2014-03-31

**Authors:** Alexandra Reichenbach, David W. Franklin, Peter Zatka-Haas, Jörn Diedrichsen

**Affiliations:** 1 Motor Control Group, Institute of Cognitive Neuroscience, University College London, London WC1N 3AR, UK; 2 Computational and Biological Learning Lab, Department of Engineering, University of Cambridge, Cambridge CB2 1PZ, UK

## Abstract

The human motor system is remarkably proficient in the online control of visually guided movements, adjusting to changes in the visual scene within 100 ms [1–3]. This is achieved through a set of highly automatic processes [4] translating visual information into representations suitable for motor control [5, 6]. For this to be accomplished, visual information pertaining to target and hand need to be identified and linked to the appropriate internal representations during the movement. Meanwhile, other visual information must be filtered out, which is especially demanding in visually cluttered natural environments. If selection of relevant sensory information for online control was achieved by visual attention, its limited capacity [7] would substantially constrain the efficiency of visuomotor feedback control. Here we demonstrate that both exogenously and endogenously cued attention facilitate the processing of visual target information [8], but not of visual hand information. Moreover, distracting visual information is more efficiently filtered out during the extraction of hand compared to target information. Our results therefore suggest the existence of a dedicated visuomotor binding mechanism that links the hand representation in visual and motor systems.

## Highlights

• Reafferent visual hand information is processed independently from attention
• In contrast, online processing of target information is facilitated by attention
• Visual hand information is filtered more efficiently than visual target information
• A dedicated visuomotor binding mechanism links visual and motor systems

## Graphical Abstract

**Figure:**
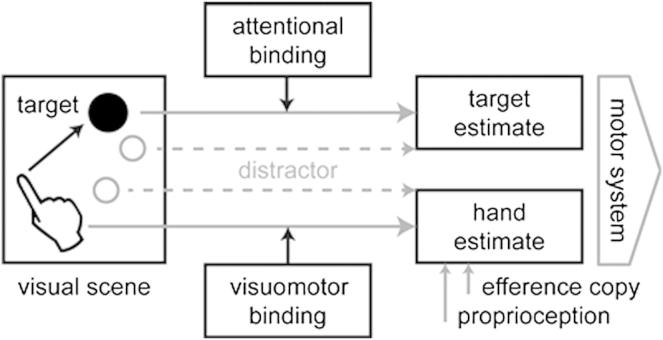

## Teaser

The brain utilizes visual information for online motor control with astonishing efficiency. Reichenbach et al. provide evidence for a specialized visuomotor mechanism that links visual hand information to corresponding motor control centers and functions independently of visual attention, which in turn promotes processing of target information.

## Results and Discussion

Skilled motor control demands the simultaneous processing of different sources of visual information. For example, when several basketball players jump for the ball at the same time, a player must track visual information pertaining to the target (exafference; the basketball) and the controlled limb (reafference; one’s own hand) while ignoring distracting visual information (e.g., other players’ hands or the background). How does the visuomotor system accomplish this task efficiently? In perceptual tasks, selection of relevant sensory information is considered to be a function of attention [7]. Likewise, one might suggest that visual attention also facilitates processing of all relevant visual information during the control of movement. Directing visual attention to the target of a reach accelerates the initiation of the movement, and overt attention (gaze direction) is therefore typically focused on this target [9, 10]. Consequently, covert visual attention would have to be allocated to the moving limbs to achieve accurate feedback control. When one reaches with both hands to two separate targets simultaneously [11], for instance, this division of resources would place high demands on a limited-capacity visual attention system [7].

**Figure 1:**
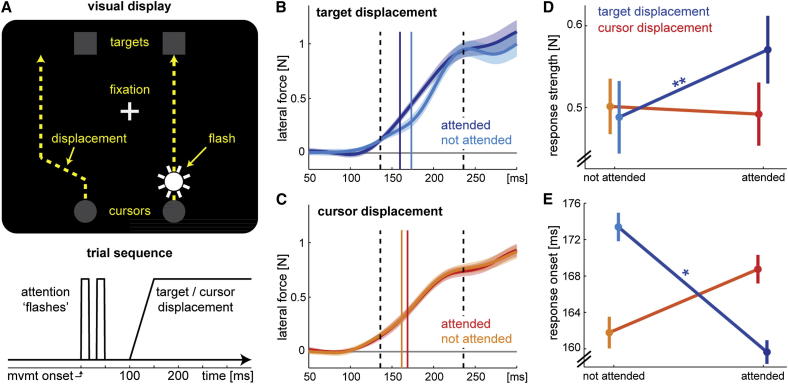
Exogenous Cuing of Visual Attention Influences Processing of Target, but Not Cursor, Information (A) Visual scene during an upward bimanual reaching trial with a displacement of the left cursor (gray circle, representing left hand position) and a flash on the right cursor. Displacements and flashes could occur on either side, i.e., the side of the flash was uninformative for the side of displacement. The same experimental manipulations (flashes and displacements) were applied in separate trials to the targets. Fixation was enforced on the central cross, which was positioned such that target and cursor displacements occurred at the same retinal eccentricity. The lower panel shows the time course of the experimental manipulations (flashes and displacements). (B) Lateral forces applied in force channels in response to target displacements on attended and unattended sides. Force traces were aligned to the onset of the displacement (time point 0 ms) and flipped such that positive forces indicate a response in the expected direction (i.e., left corrective force for a rightward cursor or leftward target displacement). Force traces were averaged across participants, and shaded areas denote 1 SEM. The solid vertical lines mark response onset, and the dashed lines the time window over which the forces were averaged to obtain the response strength (from 30 ms pre- to 70 ms postresponse onset). (C) Lateral force in response to displaced cursors analogous to (B). (D) Average response strength around response onset. Attention had a large effect [12] (*d* = 0.83) on the response strength to target perturbations (*t*_13_ = 3.116, p = 0.008), but a small effect in the opposite direction (*d* = −0.14) on the response strength to cursor perturbations (*t*_13_ = 0.516, p = 0.614). (E) Response onsets to visual perturbations. Attention had a medium-sized effect on target displacement (*d* = 0.64, *t*_13_ = 2.390, p = 0.033) and an opposite, nonsignificant effect on cursor displacement perturbations (*d* = −0.56, *t*_13_ = 2.080, p = 0.058). All error bars denote 1 SEM among participants. ^∗^p < 0.05, ^∗∗^p < 0.01.

To study the role of attention in visual feedback control, we challenged the visuomotor system with a bimanual reaching task. Participants held a robotic manipulandum with each hand and moved these simultaneously to two targets presented in the left and right visual fields while keeping their eyes fixated at a central location (enforced via eye tracking). During the task, participants’ hands were occluded by a horizontally mounted monitor, which displayed the cursors and targets (Figure 1A). The task therefore required participants to simultaneously process visual information from two targets and two cursors. To test whether spatial visual attention influences the processing of visual target and hand information during online control, we manipulated the locus of attention via exogenous cuing. Immediately after the onset of the movement, covert attention was attracted by briefly increasing the luminance [13] of a target or cursor (“flashes”; see Figure 1A). We assessed the influence of attention by displacing the position of one of the targets or cursors perpendicular to the reaching direction 100 ms after the flash [14] (Figure 1A). The side of the displacement was independent of the side of the preceding attention manipulation, but it occurred always on the same object type (target or cursor) as the attentional cue. The displacement evoked an automatic feedback response with the corresponding hand: rightward for target displacements to the right and leftward for cursor displacements to the right [1, 4, 15]. The forces with which participants pushed into “force channels” [16] during interspersed probe trials provided a sensitive assay of the early corrective motor response [1].

We found fast (onsets around 165 ms) and consistent responses to both target and cursor displacements. These indicate that the sensorimotor system, rather than relying exclusively on proprioceptive or efference copy information, is exquisitely sensitive to visual feedback from the hands [17, 18], even when it tracks both hands simultaneously. The force response to target displacements (Figures 1B and 1D) was modulated by the locus of attention. Displacements preceded by the exogenous cue elicited significantly stronger initial responses (for statistical details, see Figure 1D) and produced earlier onsets of the correction (Figure 1E) than uncued displacements. The size of the attentional modulation on feedback responses was of similar magnitude as for simple reaction time tasks [19]. In contrast, exogenous cuing did not modulate the responses to cursor displacements (Figures 1C–1E). The interaction for displacement type (target or cursor) × attention was significant for both response strength (*F*_1,13_ = 7.129, p = 0.019) and onset (*F*_1,13_ = 10.005, p = 0.008).

To corroborate these findings, we conducted a second experiment in which we manipulated covert attention endogenously using a secondary perceptual task. At the start of a trial, we presented an arrow near fixation that pointed to the left or right (cue validity for the perceptual task: 83%). During the reaching movement, the luminance of one of the targets (or, in separate blocks, the luminance of one of the cursors) was subtly changed for 350 ms. After completing the movement, participants reported whether the luminance had increased or decreased. During the movement, we probed feedback control by displacing either the left or right target (or, in separate blocks, the cursor) 100 ms before the luminance change.

**Figure 2:**
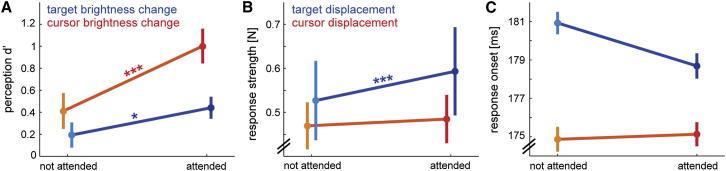
Endogenous Cuing of Visual Attention Influences Processing of Target, but Not Cursor, Information (A) Sensitivity (d′) to distinguish between brightness increases and decreases during the perception task for nonchannel trials (50% of trials), depending on whether the change occurred on the cued (attended) or noncued (not attended) side. (B) Average response strength in the force channels around response onset. Attention had a large effect (*d* = 1.02) on response strength to target perturbations (*t*_18_ = 4.43, p < 0.001), but only a small (*d* = 0.16) and insignificant (*t*_13_ = 0.69, p > 0.5) effect on the response strength to cursor perturbations. (C) Response onsets to the visual perturbations. The statistical interaction test failed to reach significance (*F*_1,18_ = 0.919, p = 0.350). All error bars denote 1 SEM among participants. ^∗^p < 0.05, ^∗∗∗^p < 0.001.

Accuracy on the brightness discrimination task was better on the cued side (*F*_1,18_ = 18.449, p < 0.001; Figure 2A), demonstrating successful attention manipulation both for the cursor and target conditions. Corrective motor responses to target displacements were also significantly greater for the attended side (Figure 2B includes statistical details). In contrast, the response to cursor displacements was not modulated by the cue. As in the exogenous case, the displacement type (target or cursor) × attention interaction was significant (*F*_1,18_ = 5.030, p = 0.038). The time of response onsets revealed a similar pattern, although here the interaction did not reach significance (Figure 2C).

These results indicate that both exogenous and endogenous visual attention modulate the processing of target information [9]. In contrast, the processing of visual information about the moving limb, although fast and efficient, seemed to be independent of either attentional manipulation. Our failure to detect an effect of the attentional modulation on responses to cursor perturbations is unlikely to be caused by a simple lack of statistical power. The power for detecting an effect of the size of the attentional modulation on responses to target displacements was >80% with our design (82.1% and 98.7% for experiments 1 and 2, respectively). Furthermore, the significant interaction shows that an attentional modulation effect was clearly larger for target displacements than for cursor displacement—if the latter was present at all. Finally, the absence of attentional modulation on cursor displacement responses is unlikely to be a result of a ceiling effect limiting the size of the maximal response to cursor displacements. In an additional control experiment (Supplemental Information available online), we introduced visual distractors alongside targets and cursors to reduce the size of the feedback response. Even though the demand on visual processing increased, additional allocation of visual attention facilitated only target, but not cursor, processing, ruling out a ceiling effect. We therefore suggest that the binding of reafferent visual information about the movement to the corresponding motor command is achieved without (or at least compared to target processing with much less) aid of visual attention. Therefore, we propose the existence of a separate visuomotor binding mechanism that confers a privileged status on visual information representing the kinematics of a moving limb.

**Figure 3:**
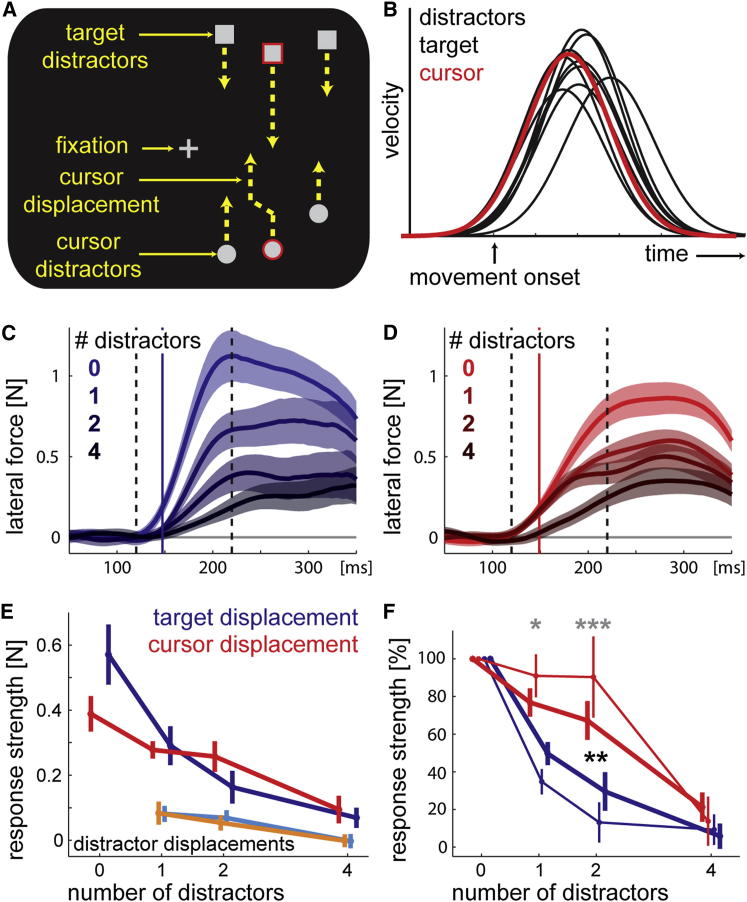
Distractors in Cluttered Visual Displays Interfere More with Target Than with Cursor Processing (A) Visual scene during an upward unimanual reaching trial with two distractors and a displacement of the cursor (red circle, representing right hand position). The target and cursor were marked red before the trial started. Zero, one, two (depicted), or four distractors were located at random positions around the cursor and target. The target and the distractors moved with a similar velocity as the cursor, such that the target was intercepted around the height of the fixation cross. Fixation was enforced on the central cross. The target, the cursor, or a distractor was displaced at each trial. (B) Example of velocity profiles for the cursor, target, and their accompanying distractors. The target and distractors moved with a minimum jerk velocity profile, with reaction and movement times sampled randomly from the participant’s reaction time and movement time distributions. The average correlation between velocity profiles of distractors with velocity profiles of the cursor or target was *r* = 0.73 ± 0.01. (C) Lateral forces applied in force channels in response to target displacements for all distractor conditions. Conventions are analogous to Figure 1. The solid vertical lines mark the response onset for the no-distractor condition, and the dashed lines the time window over which the forces were averaged to obtain the response strength (from 30 ms pre- to 70 ms postresponse onset). (D) Lateral force in response to displaced cursors analogous to (C). (E) Average response strength around response onset as a function of displacement type (target or cursor) and number of distractors. The erroneous responses to distractor displacements appear in light blue (target distractors) and orange (cursor distractors). (F) Response strength normalized to the no-distractor conditions to account for baseline differences in the responsiveness to target and cursor perturbations [20]. Thin lines indicate a small time window confined to the immediate time around the response onset (from 10 ms pre- to 20 ms postresponse onset). All error bars denote 1 SEM among participants. The black asterisks indicate significant differences between cursor and target displacements in the longer time window, the gray asterisks in the shorter time window. ^∗^p < 0.05, ^∗∗^p < 0.01, ^∗∗∗^p < 0.001.

If different mechanisms underlie attentional and visuomotor binding, they may also differ in their ability to filter out and ignore distracting objects. Due to the limited capacity of visual attention [7], filtering constitutes a problem in complex natural scenes. When reaching in such a cluttered environment, the visuomotor system faces the same challenge. To test for differences between attentional and visuomotor binding, we conducted a unimanual reaching experiment requiring participants to filter out numerous distracting objects. In order to match perceptual difficulty for the filtering of target and hand information, we presented a target that moved toward the starting position (Figure 3A; Supplemental Experimental Procedures), and participants were instructed to intercept the target with a movement of their right hand while maintaining central fixation (Figure 3A). Between zero and four distractors moved alongside the target and the cursor (the number of distractors matched for the target and the cursor). The target, target distractors, and cursor distractors followed a minimum jerk trajectory, with the onset and speed drawn randomly from the distribution of participants’ own reaching movements (Figure 3B). Given that fixation was tightly controlled, the visual information pertaining to target and hand were very closely matched.

With no distractors, responses to cursor displacements were 32% smaller than responses to target displacements (0.57 N versus 0.39 N; Figure 3E), most likely because hand estimates integrate both visual information (indicating a displacement) and proprioceptive information (indicating no displacement) [20]. Although responses to both target (Figure 3C) and cursor (Figure 3D) displacements decreased with increasing number of distractors, the response to target displacements declined much more rapidly (interaction: *F*_3,27_ = 8.571, p < 0.001; Figure 3E). Participants also showed small erroneous responses to distractor displacements. These, however, were not significantly different between perturbation conditions (main effect perturbation [target or cursor]: *F*_1,9_ = 0.041, p = 0.844; interaction: *F*_2,18_ = 0.079, p = 0.924; Figure 3E, light-blue and orange lines).

The differential performance in filtering was especially apparent after we accounted baseline responsiveness differences by normalizing the data in the distractor conditions to the corresponding data without distractors (Figure 3F). When distractors were introduced, we observed that the normalized response strength for cursor displacements remained significantly higher than did responses to target displacements (*F*_1,9_ = 19.968, p = 0.002; Figure 3F, bold lines). Interestingly, the difference in the filtering properties for target and cursor information was especially pronounced in the very early phase of the response (*F*_1,9_ = 25.843, p < 0.001; Figure 3F, thin lines). When averaged over the first 140–170 ms, the response to a cursor perturbation with up to two distractors did not decline from the level of response without distractors (*t*_9_ < 0.44, p > 0.2).

These results demonstrate that responses to cursor displacements are more robust against the presence of distracting objects than responses to target displacements. This dissociation further supports the existence of an attention-independent visuomotor binding mechanism that extracts visual information about the body. The difference in filtering efficiency can only be attributed to different processing mechanisms because we carefully matched low-level visual characteristics such as motion energy, visual hemifield, and visual eccentricity. Our result with four distractors also shows that even the filtering through visuomotor binding is resource limited—but taken together, the results indicate that these resource limitations are different from those imposed by attentional processes.

A dedicated visuomotor binding mechanism during voluntary movements would explain the efficiency and speed with which humans can execute multiple goal-directed movements at the same time [11]. This is not because visuomotor binding provides faster responses than other processes, but because its independence of visual attention frees those limited resources for allocating them to the current target during reaching [9] and to potential alternative targets or interfering objects [8]. Why the brain has developed a specialized mechanism for processing reafferent visual information, instead of relying on the general-purpose mechanisms of attention, remains to be answered by future research. We can only speculate that the necessity to react very rapidly to divergent visual information about both the target and the hand has been evolutionary important enough to justify the development of a specialized mechanism. While attention is already involved in the detection and selection of targets before movement onset [8, 9], it seems sensible that it should retain this function during online control, supporting the flexibility of the visuomotor system to adjust movement goals midreach. In contrast, the effector is rarely changed within a movement, and visuomotor binding heavily depends on efference copy and proprioceptive information. These special characteristics may have further promoted the emergence of a dedicated process.

Note that we are not claiming that processing of the target during the online control of movement is conscious or voluntary. On the contrary, there is substantial evidence that online corrections to target displacements can bypass voluntary control [4, 21] and even occur without conscious awareness [15]. The same has been shown for processing of visual hand information [1, 20]. What we show here is that this automatic target processing can be facilitated by the allocation of visual attention—whereas the processing of hand information cannot. Thus, our results demonstrate that even though processing of visual target and hand information share some features regarding their automatic and involuntary nature, the processing of visual hand information appears to occur through a dedicated channel that is uninfluenced by the allocation of visual attention.

We suggest that the visuomotor binding mechanism detects spatiotemporal correlation between objects in the visual scene and internal state estimates of moving limbs. This internal estimate is informed by proprioceptive information, and the predictions arising from a forward model through an efference copy [22] of the executed motor commands [23–25]. Visuomotor binding is complicated by the fact that the spatial relationship between movement and visual consequences is often highly task dependent. However, the ease with which we handle tools [26] or remotely controlled objects such as computer cursors [27] suggests that it is a highly flexible process that can learn new mappings between motor commands and visual outcomes. The factors influencing these adaptive processes and their relationship to the adaptation of dynamic forward models [28], however, have yet to be elucidated.

Assignment of visual input to one’s own action has been debated extensively as “intention or action attribution” or “agency” in the context of conscious perception [29, 30]. These studies show that sensory events judged to be consequences of one’s own actions are perceived differently from sensory events judged to arise from external causes [31–33]. Our results emphasize that the detection of visual stimuli pertaining to our own movements is a fundamental process for the online *control* of reaching movements. The link between actions and visual consequences allows the motor system to respond rapidly to visual feedback signaling reach errors, even in the face of potentially distracting visual information as present in naturalistic visual scenes. Visuomotor binding is most likely a phylogenetically old mechanism common to all species that rely on vision for movement guidance. It must therefore constitute a central concept in theories of visuomotor feedback control. Indeed, it is possible that this process forms the basis upon which the percept of a “sense of agency” is founded.

The proposal of a dedicated mechanism raises the question of how visuomotor binding is implemented in the nervous system. Visual attention arises through top-down influences of parietal and prefrontal areas onto sensory regions, increasing signal-to-noise in these regions through an increase in sensitivity and contrast gain of relevant neurons [34–37]. Visuomotor binding could act through a conceptually similar yet independently implemented mechanism. For example, one may hypothesize that the link is established by neural synchronization between sensory areas and corresponding motor cortical regions [38] or upstream premotor and parietal regions coding visual space near the hand [39]. Sudden sensory changes would then be directly transmitted to the respective motor circuits for rapid feedback control.

While more work remains to be done to further characterize the proposed mechanisms behaviorally and shed light on its neural implementation, our current results provide the first strong evidence for the existence of a visuomotor binding mechanism that is dissociable from general visual attention. This specialized mechanism constitutes the connecting link between sensory and motor systems by providing a privileged channel for reafferent visual information.

## Experimental Procedures

Further details about the experimental procedures and the control experiment are described in the Supplemental Information.

## Supplemental Information

Document S1. Supplemental Experimental Procedures and Figure S1
