# Supplementary material for: A Dedicated Binding Mechanism for the Visual Control of Movement
Source: Curr Biol. 2014 Mar 31;24(7):780–5. doi: 10.1016/j.cub.2014.02.030 (PMC3988841; doi:10.1016/j.cub.2014.02.030)
Supplement: Document S1. Supplemental Experimental Procedures and Figure S1 [file mmc1.pdf]

Current Biology, Volume *24*

Supplemental Information

A Dedicated Binding Mechanism for the Visual Control of Movement

Alexandra Reichenbach, David W. Franklin, Peter Zatka-Haas, and Jörn Diedrichsen

Supplemental Experimental Procedures

Participants

Neurologically healthy right-handed [S1] volunteers were recruited from an internal experiment database. All participants provided prior written informed consent and were paid for participating. They were naïve to the purpose of the experiment and debriefed afterwards. The UCL research ethics committee approved all experimental procedures.

Apparatus and visual scene

Participants were seated comfortably in front of a virtual environment setup, leaning slightly forward with their forehead supported by a chin and forehead rest. They made 20cm reaching movements away from and towards their body while holding onto a robotic manipulandum (update rate 1kHz; position and force data sampled at 200Hz) with each hand (experiments 1 and 2) or with only their right hand (experiment 3). Movements involved shoulder, elbow, and wrist in the horizontal plane at chest height. An LCD monitor (update rate 60Hz) mounted horizontally above the manipulanda prevented direct vision of the hands, but allowed participants to view the visual scene on the monitor. An eye tracker (EyeLink 1000, SR Research Ltd., Kanata, Ontario, Canada) recorded the left eye's position with 200Hz and the data was processed in real-time by the control PC to provide feedback about eye fixation (see below).

The visual display included cursor(s) indicating the hand position(s) (filled circle(s) of 0.6cm diameter), located vertically above the real positions of the hand(s). Reaching movements for each hand were executed from a start box to a target box (filled square(s), 0.6cm size, 6cm distance to the right (and left) from body midline), alternating between up- and downward movements. Fixation had to be maintained on a white fixation cross (0.5cm) located at body midline at a height such that all visual perturbations occurred at the same eccentricity from central fixation.

We applied two types of perturbation during the reaching movements. Target displacements consisted of a 2cm displacement of the visual target in the lateral direction (left or right in the x-direction), ramped over a 50ms interval. Cursor displacements consisted of a 2cm displacement of the visual cursor in the lateral direction. The necessary correction to both perturbation types was equal in size, but opposite in direction, i.e. a target displacement to the right caused a corrective response to the right while a cursor displacement to the right caused a corrective response to the left. Both perturbations could be easily detected and participants were informed about their occurrence before the experiment started. However, responses to such perturbations are highly automatic and immune to voluntary processes [1, 4, S2].

Eye fixation control

The position(s) of the fixation cross(es) in eye coordinates were obtained from each participant with a calibration procedure before the start of the experiment. To avoid frequent re-calibration or problems due to drift or head movements, we used a combined manual / automatic procedure to ensure eye fixation throughout a trial. During the training, the experimenter checked the mean eye position of trials reported as valid (obtained from the automatic procedure, see below) for discrepancies between expected and actual mean eye position. Participants fixating elsewhere were reminded to keep their eyes on the fixation cross. During the experiments, we relied on the automatic procedure, but sample inspection ensured that the participants did not change their strategy and fixated somewhere else. The automatic procedure required that 80% of the recorded eye tracking data within the movement phase of a trial was technically valid (i.e. at most 20% missing values received from the eye tracker). From the validly recorded data, 68.2% was required to be within 1mm (in screen coordinates) of the mean eye position of the trial. Trials not fulfilling these criteria were automatically rejected as invalid eye fixation trials, and repeated. Because eye fixation needs a visual anchor to be stable [S3], we can exclude the possibility that the eyes rested on the blank screen adjacent to the fixation cross or moved around if a trial was accepted by the automatic procedure.

General procedure

Participants initiated a trial by moving the cursor(s) into the start while maintaining eye fixation. After 350ms, the target(s) appeared at 20cm distance from the start positions. Participants were instructed to initiate fast and accurate reaching movements toward the target(s) when the fixation cross changed shape (two small circles were added centrally over the fixation cross), which happened 2.3s after the target(s) appeared. The trial ended when hand velocity dropped below 3.5cm/s for at least 40ms. A trial was considered valid when eye fixation was maintained, movement duration was shorter than 800ms, and maximum velocity ranged between 50 and 80cm/s. Valid trials with endpoint accuracy of at least 7mm were rewarded with one point per hit target, an animated "explosion", and a pleasant tone. A running score was displayed at the top of the screen. Feedback about trial performance (accuracy / velocity / eye fixation) was given via a color scheme at the end of each trial. Participants were encouraged to use this visual feedback to adjust their performance. Invalid trials constituted 15%/10%/20% of all executed trials (experiment 1/2/3) and were repeated by randomly intermixing them into the remaining trials of the current experimental block.

In half of the trials, a "force channel" restricted movements, guiding the hands on a straight path to the targets. The force channel was implemented with a spring-like force of 7000N/m applied in the lateral direction. The force with which participants pressed into the channel provided a more sensitive assay of the feedback triggered responses than position data from unconstrained trials [1, 15]. The sensitivity is similar to velocity data but as force is measured directly, and not differentiated, no additional noise is introduced. On channel trials, the target or cursor was displaced back after 350 ms to enable participants to reach the target. On non-channel trials, the target and cursor displacements remained, requiring participants to correct for the perturbations. We refrained from using force channel trials during training blocks in order to avoid a possible attenuation of the feedback response [1].

Experiment 1: Exogenous cueing of attention

Fourteen participants (8 female, 23.4±3.8 years) completed one experimental session (~1.5 hours). After one training block, they performed 8 experimental blocks of 80 trials. Each block consisted of the randomized permutation of all experimental conditions: perturbation type (target/cursor) x movement (up-/downwards alternating) x channel (y/n) x flash side (left/right hemi-field) x 5 displacement conditions. Four different displacements (left/right hemi-field x leftward/rightward) and one condition without displacement were tested. The cursors, start and target boxes were dark grey throughout the trials. When the mean tangential velocity of both hands exceeded 3.5cm/s ("mvmt onset" in Fig. 1a), the corresponding object (cursor or target) alternated its color between white and gray twice within 50ms ("flash" in Fig. 1a). This exogenous cue was presented on the left or right cursor on blocks with cursor displacements, and on the left or right target on blocks with target displacements. The displacement occurred 100ms after triggering the flashes, which corresponded to roughly 5cm into the movement.

Experiment 2: Endogenous cueing of attention

Nineteen participants (13 female, 24.0±4.7 years) completed a pre-test and two experimental sessions (~2 hours each). During the pre-test, either one cursor or one target changed its brightness for 350ms during the reaching movement. Participants' task was to decide after the movement whether luminance had increased or decreased during the reach (2 alternative forced choice task). By using different levels of brightness change, we determined a contrast level that yielded a perceptual performance of d' = 0.3 (separately for cursors and targets) for each individual participant, which was then used throughout the experiment. One additional participant was pre-tested but excluded from the experiment because of chance performance up to the highest contrast level.

The experiment consisted of 16 blocks of 48 trials. The site of color change and displacement (cursor vs. target condition) alternated between blocks. Thus, participants could concentrate on either the

cursors or targets for the perceptual task. Each block contained 50% non-channel trials used for assessing perceptual performance. These consisted of a randomized permutation of all experimental perception conditions: movement (up-/downwards, alternating) x cuing (left/right) x brightness change (1/3 incongruent, 2/3 congruent with cue) x change direction (brighter/darker). The remaining 50% were channel trials, used for assessing fast feedback responses to displacements, with the randomized permutation of all experimental reaching conditions: movement (up-/downwards, alternating) x cuing (left/right; brightness change was always congruent with the cue) x side of displacement (left/right) x direction of displacement (left/right/none). The cursors, start and target boxes were medium grey throughout the trials. An arrow pointing left- or rightwards adjacent to the fixation cross served as central cue. In perturbation trials, the displacement occurred when both hands had moved an average 5cm in the forward direction. The brightness change occurred 100ms after the displacement (or the point in time when a displacement would have occurred for unperturbed trials) such that it could not interfere with the early response to the displacement. After each successful reach, participants made the perceptual judgment.

Experiment 3: Distractor interference in a complex visual scene

Ten participants (5 female, 22.0±2.8 years) completed two experimental sessions (~1.5 hours each). After four training blocks (up-/downwards without distractors, up-/downwards with distractors), they carried out 20 experimental blocks of 76 trials. Two additional participants were trained but excluded from the second day of the experiment because they performed poorly (best block fewer points than the average block score). The target movement ended in the middle of the screen at about the height of the fixation cross. Participants had the goal to terminate their cursor movement as close to the target endpoint as possible (Fig. 3a). In order to maintain the length of the reaching movement at 20cm while still using the same visual field as in the first two experiments, we compressed the visual scene by factor 2 in the y-direction. Therefore, both cursor and target moved about 10cm visually while the hand moved 20cm physically. At the height of the start box, the cursor was located vertically above the hand. Thus, we alternated between up- and downward blocks to

keep the visuo-motor mapping constant within a block. The order of up- and downward blocks was counterbalanced across participants. Even though adaptation to a constant visuo-motor gain mapping happens virtually instantly and generalizes across directions [S4], we started each block with four unperturbed and undistracted movements to allow for adaptation. The remaining 72 trials of each block consisted of the randomized permutation of all experimental conditions: perturbation type (target/cursor) x channel (y/n) x displacements (18 conditions). Without distractors, there were 3 displacement conditions (to the left/right/none). With distractors (1, 2, or 4), there were 5 displacement conditions (cursor/target to the left or right, distractor to the left or right, or none).

The cursors, start and target boxes were white throughout the trials. In distractor trials, the distractors appeared together with the target. The distractor starting positions were uniformly distributed around the target/cursor initial positions, ±4cm horizontally and ±2cm vertically, with a minimum horizontal distance of 1.2cm. The target and the distractors moved with a minimum jerk profile in y-direction [S5] while the x-position remained constant. The onset time and movement time for each target and distractor were sampled independently from the measured distribution of the RT and MTs of each individual participant (Fig. 3b). Thus, the motion of the target and distractors mimicked the cursor motion, but their velocity profiles in y-direction correlated only partially with that of the target or cursor ($r=0.73\pm0.01$). The actual cursor and target had a red border from target appearance until the cue to initiate the movement. In perturbation trials, the displacement occurred when the corresponding hand had moved 2cm in the forward direction.

Statistical analysis

As invalid trials were repeated within each block, we averaged over 8/8/10 repetitions (experiment 1/2/3) for each condition and participant. All position and force traces were aligned temporally to the onset of the visual perturbations, or, for unperturbed trials, the point in time when the perturbation would have occurred. The measured lag of 50ms between commanded visual change and the real visual change on the screen due to processing time in the graphical output and the screen refresh rate was taken into account for the analysis.

To assess corrective reaching responses, we measured the lateral forces exerted into the channels (perpendicular to the reaching direction, cf. Fig. 1b,c, 3c,d). Response onsets (Fig. 1b,c,e, 2c, 3c,d) for each subject and condition were determined by performing t-tests between the force traces of all leftward and rightward corrections until at least 4 consecutive tests revealed significant differences ($p < .05$). The time stamp of the first of those 4 consecutive tests was taken as response onset [S6, S7]. For all further analyses, we mirrored the force traces for which we expected a negative force response and then averaged over perturbation directions. This automatically removed any constant force profiles caused by the biomechanical properties of the arm and robot. Furthermore, we pooled the data for the conditions of no interest, namely up- and downward movements, and the right and left hand for the first two experiments. To obtain a time-averaged single measure for the early response strength, we averaged the forces around mean onset time across no distractor conditions and participants (from 30ms before until 70ms after response onset, Fig. 1b,c, 3c,d).

For statistical assessment, we used repeated-measures ANOVA (within each experiment), and two-tailed t-tests between conditions (paired where applicable). Corrections for multiple comparisons were performed using Bonferroni corrections where necessary.

Supplemental Control Experiment

To exclude the possibility that the lack of attention effect on the processing of hand information resulted from a ceiling effect, we conducted a control experiment replicating Experiment 1. Within this experiment, we tested an additional control condition, in which we suppressed the overall responsiveness to the visual displacements by introducing a distractor for each target and each cursor. The movement of the cursor distractors was implemented in the same manner to those in Experiment 3, and the target distractor were stationary at the same spatial distance as the cursor distractors. The overall response strength was indeed decreased by introducing distractors (Fig. S1). In fact, the responses to target and cursor displacements were suppressed to similar amounts as expected for very complex scenes comparable to the 4-distractor condition in Experiment 3. The response pattern regarding the attention manipulation, however, replicated our previous results from Experiments 1 and 2: Only the force response to target displacements was modulated by the locus of attention. Displacements preceded by the exogenous cue elicited significantly stronger initial responses (for statistical details see Fig. S1) than displacements that were uncued. In contrast, exogenous cuing did not modulate the responses to cursor displacements for any distractor condition. This is corroborated by the significant interaction ($F_{1,8}$=8.554; p=.019) for displacement type (target/cursor) x attention, whereas the three-ways interaction also including distractors (without/with) was not significant ($F_{1,8}$=0.261; p=.624). These results clearly show that processing of visual hand information is not modulated by visual attention, even when there is the possibility that it increases the response strength in the presence of distractors, as shown in the target condition.

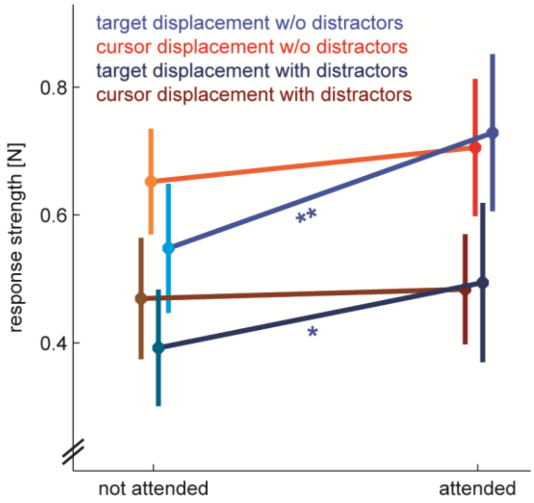

Figure S1 **Responses to target and cursor perturbations in the control experiment.** Related to “Results and Discussion”. Attention had a significant effect on the response strength to target perturbations (without distractors: t_8=4.744; p<.001; with distractors: t_8=2.448; p=.020), but no effect on the response strength to cursor perturbations (both p>.1). Error bars denote 1 SEM between participants. * p<.05, ** p<.01.

Supplemental References

S1. Oldfield, R.C. (1971). The assessment and analysis of handedness: The Edinburgh inventory. Neuropsychologia *9*, 97-113.

S2. Day, B.L., and Brown, P. (2001). Evidence for subcortical involvement in the visual control of human reaching. Brain *124*, 1832-1840.

S3. Cornsweet, T.N. (1956). Determination of the stimuli for involuntary drifts and saccadic eye movements. J Opt Soc Am *46*, 987-993.

S4. Krakauer, J.W., Pine, Z.M., Ghilardi, M.F., and Ghez, C. (2000). Learning of visuomotor transformations for vectorial planning of reaching trajectories. J Neurosci *20*, 8916-8924.

S5. Todorov, E., and Jordan, M.I. (1998). Smoothness maximization along a predefined path accurately predicts the speed profiles of complex arm movements. J Neurophysiol *80*, 696-714.

S6. Oostwoud Wijdenes, L., Brenner, E., and Smeets, J.B. (2013). Analysis of methods to determine the latency of online movement adjustments. Behav Res Methods.

S7. Reichenbach, A., Costello, A., Zatka-Haas, P., and Diedrichsen, J. (2013). Mechanisms of responsibility assignment during redundant reaching movements. J Neurophysiol *109*, 2021-2028.
